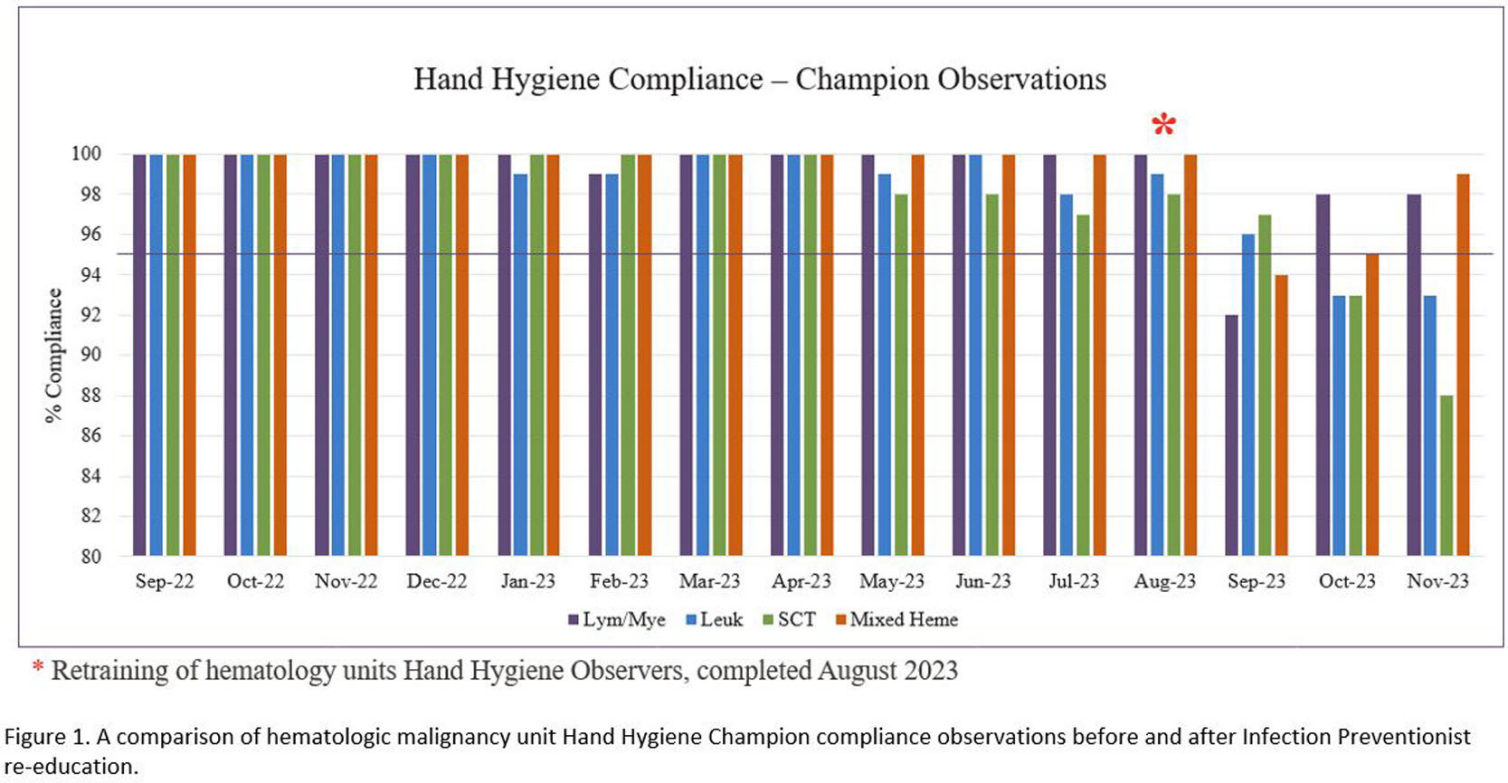# Improving Data Quality from a Hematology Unit Hand Hygiene Observation Program

**DOI:** 10.1017/ash.2024.246

**Published:** 2024-09-16

**Authors:** Adina Feldman, Sherry Cantu, Hilary McMurry, Crystal Odom, Leila Nahavandi, Jane Powell, Amy Spallone

**Affiliations:** MD Anderson Cancer Center; The University of Texas MD Anderson Cancer Center; The University of Texas MD Anderson Cancer Center

## Abstract

**Background:** For United States healthcare programs to be fully compliant with Joint Commission National Patient Safety Goal (NPSG) #7, organizations must implement and maintain a hand hygiene (HH) program that follows either the current Centers for Disease Control and Prevention (CDC) or the current World Health Organization (WHO) HH guidelines. Joint Commission standard IC.03.01.01 requires these organizations to provide metrics that evaluate the effectiveness of their program and program goals. Our center utilizes the direct observation method with the use of over 550 Hand Hygiene Observers (HHO) to collect our HH compliance. HHO are trained with a computer-based course that requires passing a post-education test. During fiscal year 2023 (FY23), Infection Control surveillance noted an increase in hospital-acquired infections (HAI) Clostridioides difficile infections (CDI), catheter-associated urinary tract infections (CAUTI), and multidrug-resistant organisms (MDRO) on our hematologic malignancy units (HM), which initiated an Infection Control (IC) investigation into possible causes. Increased rounding by our Infection Preventionist (IP) observed that HH compliance was much lower than unit HHO reported rates. Inquiries into this data discrepancy revealed barriers to accurate reporting, including HHO having low confidence in identifying and reporting non-compliant behavior. To that end, we conducted mandatory re-training of all HM HHO with the primary goal of improving the quality of our HH compliance data and addressing barriers with non-compliance reporting. Our secondary goal was to identify areas of improvement in institutional HH rates. **Methods:** In August 2023, 252 HM staff and HHO received detailed, in-person retraining by the HM IP. Training included reviewing the discrepancy in HHO and IP observations, potential causes of discrepancy, most commonly missed HH opportunities, examples of correct and incorrect HH practices, and addressing staff questions. **Results:** Following mandatory re-training of HM HHO, HH compliance for our HM units from September 2023 – December 2023 ranged from 89% to 98%, with increased reporting of non-compliance (Figure 1). A detailed dashboard was created that focused on HM HH compliance, containing the HHO observations and non-compliant reports. **Conclusion:** A one-time in-person retraining of HM HHO by our IP has led to an improvement in data quality, which is imperative for future quality improvement initiatives. Improving our HH data quality allowed IC to identify and provide actionable feedback to HM leaders, create targeted interventions to improve HAI rates, and improve patient safety. Future goals include retaining of all HHO and a HH campaign to ensure patient safety across our institution.

**Figure:**